# Long-term nitrogen fertilization decreased the abundance of inorganic phosphate solubilizing bacteria in an alkaline soil

**DOI:** 10.1038/srep42284

**Published:** 2017-02-09

**Authors:** Bang-Xiao Zheng, Xiu-Li Hao, Kai Ding, Guo-Wei Zhou, Qing-Lin Chen, Jia-Bao Zhang, Yong-Guan Zhu

**Affiliations:** 1 Key Laboratory of Urban Environment and Health, Institute of Urban Environment, Chinese Academy of Sciences, Xiamen 361021, China; 2 University of Chinese Academy of Sciences, Beijing 100049, China; 3 Department of Plant and Environmental Sciences, University of Copenhagen, Frederiksberg 1871, Denmark; 4 State Key Lab Soil & Sustainable Agriculture, Institute of Soil Science, Chinese Academy of Sciences, Nanjing 210008, China; 5 State Key Laboratory of Urban and Regional Ecology, Research Center for Eco-Environmental Sciences, Chinese Academy of Sciences, Beijing 100085, China

## Abstract

Inorganic phosphate solubilizing bacteria (iPSB) are essential to facilitate phosphorus (P) mobilization in alkaline soil, however, the phylogenetic structure of iPSB communities remains poorly characterized. Thus, we use a reference iPSB database to analyze the distribution of iPSB communities based on 16S rRNA gene illumina sequencing. Additionally, a noval *pqqC* primer was developed to quantify iPSB abundance. In our study, an alkaline soil with 27-year fertilization treatment was selected. The percentage of iPSB was 1.10~2.87% per sample, and the dominant iPSB genera were closely related to *Arthrobacter, Bacillus, Brevibacterium* and *Streptomyces*. Long-term P fertilization had no significant effect on the abundance of iPSB communities. Rather than P and potassium (K) additions, long-term nitrogen (N) fertilization decreased the iPSB abundance, which was validated by reduced relative abundance of *pqqC* gene (*pqqC*/16S). The decreased iPSB abundance was strongly related to pH decline and total N increase, revealing that the long-term N additions may cause pH decline and subsequent P releases relatively decreasing the demands of the iPSB community. The methodology and understanding obtained here provides insights into the ecology of inorganic P solubilizers and how to manipulate for better P use efficiency.

Phosphorus (P) is essential to the productivity of ecosystems, while alkaline soil with high pH limits P acquisition by plants and thus decreases plant diversity^1^. In agro-ecosystems, P fertilization could improve soil nutrient status. However, the applied P is easily fixed by soil and becomes unavailable for crop growth^2^. Hence, P applications greater than crop’s demand were frequently used to saturate the strong sorption of soil, increasing our dependence to this non-renewable resource^3^. Additionally, the excessive P applications in soil would be lost through leaching into surface waters, which may cause pollutions to surrounding environments^2^.

Soil microbes are important contributors in plant acquisition of P^4^. Inorganic phosphate solubilizing bacteria (iPSB) are considered as the major participant in facilitating P release via organic anion or proton secretion while phytase, phosphatase and phosphonatase are responsible for organic P degradation^5^,^6^. Previous researches have indicated that iPSB are abundant in alkaline environments^7^, yet the complete structure of iPSB communities has not been reported. The composition of organic phosphate solubilizing bacteria community have been deciphered since a universal primer targeting the *phoD* gene, encoding for alkaline phosphatase expression, was successfully developed and applied^8^. However, it is difficult to find a homologous gene among all iPSB regulating inorganic phosphate solubilization. Recently, cofactor pyrroloquinoline-quinone (Pqq) was suggested as an important regulator involved in inorganic P release and the expression of *pqq* genes could catalyze the formation of gluconic acid by the direct oxidation pathway with inorganic phosphate solubilization ability^9^. A *pqqC* primer as molecular marker for iPSB has been designed^10^, however, so far has only seen in Pseudomonads. The narrow specificity of primers limited the analyses of complete iPSB communities.

As a slow process of soil development, the bacterial community needs a long period to reach structural homeostasis. Long-term fertilization change the soil nutrient contents and thus alter the composition of bacterial communities. Nitrogen (N) fertilizer application promotes crop yield, but the low use-efficiency together with intensive application leads to N residues, threatening soil quality^11^,^12^. The negative effect of long-term N inputs on soil microorganisms has been extensively reported, including decrease of microbial biomass and respiration, weakening of plant-microbe interactions, and losses of biological diversity^13^,^14^,^15^. Beyond that, the abundance of functional bacterial groups, such as N-fixing bacteria, ammonia oxidizing bacteria and arbuscular mycorrhizal fungi (AMF), decreased significantly after long-term N fertilization^16^,^17^,^18^. However, as an important P-relating functional bacteria in soil P cycling, the response of the iPSB community to long-term fertilization has not been reported due to the lack of adequate evidence.

In this study, we hypothesized that long-term N fertilization would decrease the abundance of iPSB community in an alkaline soil. To verify this assumption, an alkaline soil with 27-year chemical and organic fertilization regimes was selected. To comprehensively analyze the iPSB community structure, we used an alignment method based on a reference iPSB database and 16S rRNA gene illumina sequencing. Additionally, for more accurately quantifying the iPSB abundance, we developed a new *pqqC* primer with a broad species range in our research. The phylogenetic structure of the iPSB community and relative abundance of *pqqC* gene was first time reported in this study.

## Materials and Methods

### Study site and soil sampling

The long-term experiment sites locate at Fengqiu Agro-ecological Experimental Station, Chinese Academy of Sciences, Fengqiu County, Henan Province, China (35°00′N, 114°24′E). The field experiment was conducted in an alkaline field (average pH_H2O_ is 8.65, and average Ca^2+^ concentration equals to 47.94 mg g^−1^) where wheat (*Triticum aestivum* L.) was grown in winter and maize (*Zea mays* L.) was cultivated in summer. Seven treatments with four replicates (28 plots) in completely randomized blocks were established^19^. The treatments were: (1) control without fertilizer, (2) mineral N and potassium (K) fertilizer (NK), (3) mineral PK fertilizer (PK), (4) mineral NP fertilizer (NP), (5) mineral NPK fertilizer (NPK), (6) organic manures (OM) and (7) combined application of half mineral NPK fertilizer and half organic manure (1/2OMN). In September 2015 (during the maize season), 5 cores of surface soil (0–15 cm) were collected in each plot. The samples were air-dried and sieved to 2 mm to remove aboveground plant residues, and stones, and stored in polyethylene bags at 4 °C. Each sample was divided into two portions for biological and chemical analyses, respectively. The detailed experiment design and fertilizer application listed in Table S1. The maize yield was provided by colleagues from Fengqiu Agro-ecological Experimental Station.

### Chemical and biological analyses

Soil pH was measured through a suspension sample with a soil (air-dried) to water (w/w) ratio of 1:2.5^20^. Total carbon (C_*Tol*_) nitrogen (N_*Tol*_) was measured with an elemental analyzer (vario max CN, Elementar, Hanau, German). A flow injection analyzer (QC8500, Lachat, Loveland, USA) was employed to analyze the concentrations of ammonium (N_*Amo*_) and nitrate/nitrite (N_*Ntr*_) within one week. The total and available phosphorus (P_*Tol*_ & P_*Osl*_) were determined by the molybdate-blue method^21^ after strong acid digestion^22^ or sodium bicarbonate extraction^23^, respectively. The acid digested samples were further used to analyze the total potassium (K_*Tol*_) concentration by inductively coupled plasma optical emission spectrometry (ICP-OES) (Optima 7000DV, Perkin Elmer, Massachusetts, USA). Microbial biomass C was determined with the chloroform fumigation extraction method within 24 h^24^.

### Genomic DNA extraction, illumina sequencing and data analysis

Genomic DNA was extracted from 0.5 g soil per sample using a FastDNA^®^ Spin Kit for Soil (MP Biomedicals, Santa Ana, California, USA) and stored at −20 °C according to the manufacturer’s instructions. DNA quality was assessed by NanoDrop ND-2000 Spectrophotometer (Thermo Scientific, Waltham, Massachusetts, USA) with OD_260_/OD_280_ ranging from 1.6 to 1.8. The concentration of DNA was determined with QuantiFluor^®^ dsDNA system (Promega, Madison, Wisconsin, USA) using multiscan spectrum (SpectraMax M5, Molecular Devices, Shanghai, China).

The amplification of bacterial fragments for illumina sequencing used primers 515F/907R, in which each reverse primers was fused with a unique 6-mer barcode for each sample^25^. PCR reactions were prepared in 50 μL reaction volumes with 1 μL *Premix Ex Taq* Hot Start Version (TAKARA, Dalian, China), 0.2 μM each primers and 0.1 mg/mL bovine serum albumin (BSA), following with PCR protocol: initial denaturation at 95 °C for 5 min, 35 cycles of 95 °C for 30 s, 58 °C for 30 s, 72 °C for 30 s and a final 5-min extension at 72 °C. The amplicons were purified with Universal DNA Purification Kit (TIANGEN, Beijing, China), quantified with QuantiFluor^®^ dsDNA system as described above, pooled at the equal molarity and submitted for sequencing using Illumina Hiseq2500 platform (Novogene, Beijing, China).

The returned raw reads were filtered, processed and analyzed using QIIME^26^. Sequence processing quality control was done by default and operational taxonomic unit (OTU) was clustered at 3% dissimilarity cutoff by UCLUST clustering^27^. RDP classifier was used to retrieve and classify representative sequences^28^. Further analysis was processed with R version 3.2.3 (The R Foundation for Statistical Computing, Vienna, Austria) with VEGAN, BIODIVERSITY and LABDSV packages. All sequences were submitted to National Center for Biotechnology Information Sequence Read Archive with the Accession no. SRP072392.

### iPSB database and sequence alignment

A reference database of iPSB was constructed by collecting 154 iPSB strains from publications based on their 16S rRNA sequences (Table S2). The Local Blast 2.2.27+ (ftp://ftp.ncbi.nlm.nih.gov/blast/executables/blast+/2.2.27/) was used to make alignment between 16S rRNA illumina sequencing data and iPSB database. The potential iPSB species were then identified and annotated with critical criteria (E-value < 1 × 10^−10^ and sequence identity >99%).

### Primer design, validation and clone library construction of pqqC

To extend taxonomic range of *pqqC* gene detection, a specific primer pair (Fw: AAC CGC TTC TAC TAC CAG, Rv: GCG AAC AGC TCG GTC AG) was designed for *pqqC* gene amplification according to consensus sequences (Figure S1) and products were predicted to range from 297 to 321 bp. PCR reaction system and protocols were the same as 16S rRNA gene amplification (without BSA) in 50 μL reaction mixtures.

To validate specificity of this primer pair, 76 iPSB strains were used (Table S3), which had been isolated from two cropland soils. These two samples were derived from an alkaline soil (pH_H2O_ = 7.70) in Heilongjiang, China (47°26′ N, 126°38′ E) and an acid soil (pH_H2O_ = 5.24) in Hunan, China (28°14′ N, 116°54′ E) (unpublished), aiming to cover diverse iPSB genera under different pH environment. To determine the inorganic phosphate solubilizing potentials, 72 iPSB strains were cultured in a modified Pikovskaya medium (PVK)^29^ without yeast extract and agar at 200 rpm and 37 °C for 72 h. After centrifuging the cultures at 12, 000 rpm for 10 min, the supernatants were used to determine the orthophosphate concentration by molybdate-blue method^21^ with sterilized PVK medium as the blank.

To further verify the coverage of *pqqC* primer, DNA of the above two soil samples were used as templates to make clone library. The DNA extractions method was the same as Genomic DNA extraction method described in the next section and PCR protocols were the same as described above. The amplified products were purified and cloned into the pMD 19-T Vector (TAKARA, Dalian, China), followed by transformation into DH5α competent cell (TAKARA, Dalian, China). The positive sub-clones (30 per sample) with ampicillin resistance were selected and submitted for sequencing (Invitrogen, Shanghai, China). By removing reads with low base calling and frame-shift errors, 52 valid sequences (accession number: KU678336~KU678387) were obtained and clustered into OTUs with 97% similarity using the Mothur program^30^. Finally, the assigned 40 sequences were translated into amino acids for pair-wise alignment and phylogenic tree construction (Figure S2).

### Quantification of pqqC gene

Real-time absolute fluorescence quantitative detection of *pqqC* gene was conducted using 16S rRNA (F515/R907) gene as ref. 31. DNA from each sample was prepared in triplicate and performed in LightCycler 480 System (Roche, Basel, Switzerland) according to the manual. The PCR reaction used for both genes were: 98 °C for 3 min for 1 cycle; and 98 °C for 20 s, followed by 62 °C for 30 s for 40 cycles. The reaction mixture consisted of 0.3 μM each primer, 10 ng template DNA, 1 × SYBR premix Ex *Taq*, 0.1 mg mL^−1^ BSA. The controls without template were added in each reaction. Standard plasmids respectively carrying with the *pqqC* and 16S rRNA gene respectively were generated by transforming both genes into vectors using pMD 19-T vector cloning kit (TAKARA, Dalian, China) and extracted using a TIAN prep mini plasmid kit (TIANGEN, Beijing, China). The concentration of plasmid DNA was measured by NanoDrop ND-2000 Spectrophotometer (Thermo Scientific, Waltham, Massachusetts, USA) for gene copy numbers calculation. Serial dilutions of standard plasmid DNA with 10-fold was conducted to make calibration curves. A dissociation curve indicated only one peak at a melting temperature (*T*_*m*_) of 91.8 °C was detected. The reactions with 90% to 110% efficiencies were accepted. Relative abundance of *pqqC* was considered as the ratio of *pqqC* against 16S rRNA gene copy number. The standard curve and typical log-transformed fluorescence curves were given in Figure S3.

### Statistic analyses

Correlation and variance (ANOVA) analysis were conducted using IBM SPSS Statistics 21.0. Figures were generated using SigmaPlot 12.5 or Microsoft Excel 2015. The sequence alignment, phylogenetic tree creation and annotation were processed with ClustalX 2.0 (http://www.clustal.org/)^32^, MEGA 6.0 (http://www.megasoftware.net/)^33^ and iTOL v3 (http://itol.embl.de/)^34^, respectively. Principal component analysis (PCA), canonical correlation analysis (CCA), variation portioning analysis (VPA), Adonis test and Monte Carlo test were performed as described previously^28^.

## Results

### Impacts of long-term fertilization on soil properties

Results showed that long-term fertilization decreased soil pH value, but increased soil nutrient contents (Table 1). Except the NK treatment, the content of C_*Tol*_, N_*Tol*_, N_*Amo*_, and N_*Ntr*_ increased significantly after fertilization, while K_*Tol*_ showed no significant difference between all treatments. Additionally, the P_*Tol*_ and P_*Osl*_ gradually increased over 27-years of fertilization except for the NK treatment (Table S4).

PCA of soil chemical properties (Figure S4) indicated that the ordination patterns of soil chemical properties have been changed by long-term fertilization, as they were well separated from the control samples. About 66% of total variance was explained by the first two axes with 39% and 27% explanations in PC1 and PC2, respectively. Samples from NK and PK were well separated from others; while treatments with N and P fertilizations, including NP, NPK, OM and 1/2OMN samples, were gathered closely.

### Phylogenetic structure of the iPSB community

In total, 2,294,188 sequences were obtained from 16S rRNA gene illumina sequencing results. To accurately estimate iPSB populations, 154 known iPSB strains, which belong to Actinobacteria, Bacteroidetes, Firmicutes and (α-, β, and γ-) Proteobacteria phyla, were collected from numerous studies as reference database for species assignment with 16S rRNA sequencing data (Table S2). Overall, the sequences highly identical to the know iPSB accounted for 1.10–2.87% of all bacterial 16S rRNA sequence reads.

**Figure 1:**
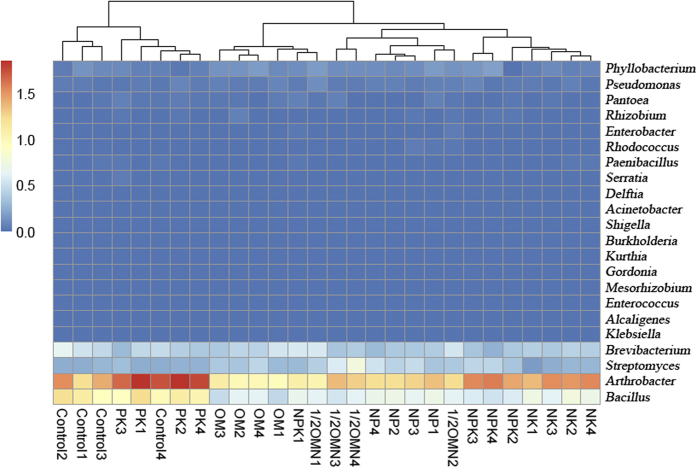
The distribution profile of iPSB abundance with different fertilizations. The sample name was labeled under each column and numbers represented sample replicates (four replicates for each treatment). The plotted values are the natural logarithm transformed proportion of iPSB abundance. The columns were clustered based on Bray-Curtis distance.

To better illustrate the phylogenetic structure of iPSB communities, the counts of iPSB species were integrated into genus level (The counts at species level were presented in Table S5). The iPSB percentages of different treatments were plotted in Fig. 1. Based on Bray-Curtis distance, the iPSB community compositions of different treatments were well separated with each other except one 1/2OMN and another control sample. In total, 22 iPSB genera were aligned and most dominant genera were *Arthrobacter* (41.1~67.4%), *Bacillus* (12.1~35.7%), *Brevibacterium* (5.2~20.6%) and *Streptomyces* (3.1~21.8%). The ratio of these four genera together was accounted for, on average, more than 90% of iPSB population except one sample from 1/2OMN treatment (70.95%), while the percentage of other iPSB genera varied among treatments.

### Assessment of pqqC primer

According to conserved sequences of *pqqC* from 9 common iPSB species, the amino acid sequences NR(F/Y)(Y/H)YQ and LTEL(F/A)(A/S) were used to design specific forward and reverse primer, respectively (Figure S1). To investigate the coverage of this primer pair, a clone library was constructed selecting two soils of different types as DNA templates. Results showed that common iPSB belonging to Actinobacteria, α-, β-, γ- Proteobacteria and Firmicutes were detected as well as an unusual iPSB phylum Verrucomicrobia (Figure S2). The specificity validation of this primer by isolated iPSB strains (Table S3) showed that most iPSB could be detected except for *Bacillus megaterium* (Firmicutes) and a few Actinobacter strains. In conclusion, the *pqqC* primer could be used to detect most iPSB strains.

### Impact of long-term fertilization on the iPSB community and pqqC gene

**Figure 2:**
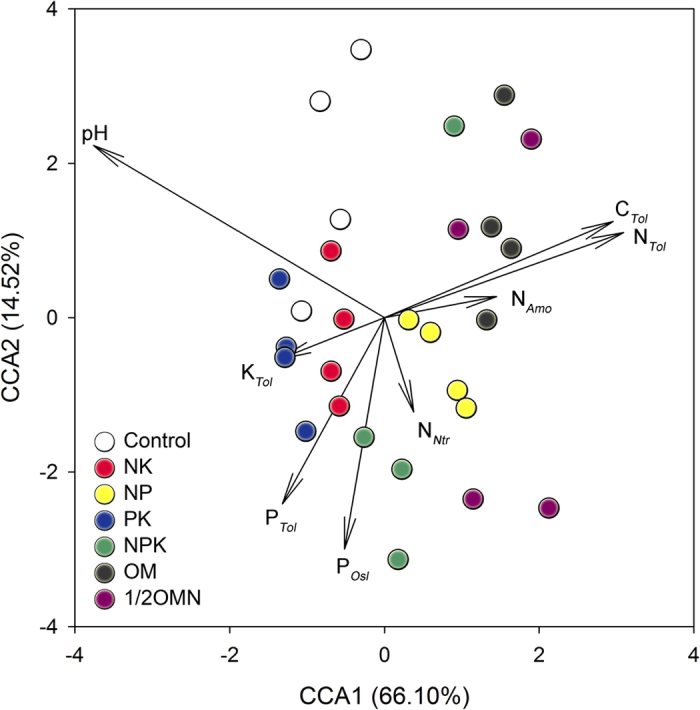
Canonical correlation analysis (CCA) of the effect of soil chemical properties (arrows) on the iPSB community (symbols) based on Bray-Curtis distance. The control, NK, NP, PK, NPK, OM and 1/2OMN treatments were indicated by white, red, yellow, blue, green, black, and purple circles, respectively. Environmental variables were shown by arrow lines. The percentage of explanation variation is shown in each axis, and the relationship is significant (*P* = 0.005).

Long-term fertilization affected the iPSB community structure. CCA results showed that treatments of the control, NK, NP, PK and OM were separated from each other (Fig. 2). Soil chemical properties totally explained 80.6% of the iPSB community structure in first two axes, and pH, C_*Tol*_ and N_*Tol*_ best explained the differences between treatments, which was validated by Monte Carlo permutation tests at *P* < 0.001 (Table 2).

N rather than P or K fertilization significantly affected the iPSB community structure and abundance. VPA results showed that N explained 20.02% of the iPSB community structure, while P and K explained 8.21% and 3.67%, respectively (Figure S5). Adonis tests (Table 3) showed that the iPSB community structures of NK and NP treatments rather than PK were different from the control at *P* < 0.05. NPK treatment was significantly different from PK samples, but similar to NK and NP treatment.

**Figure 3:**
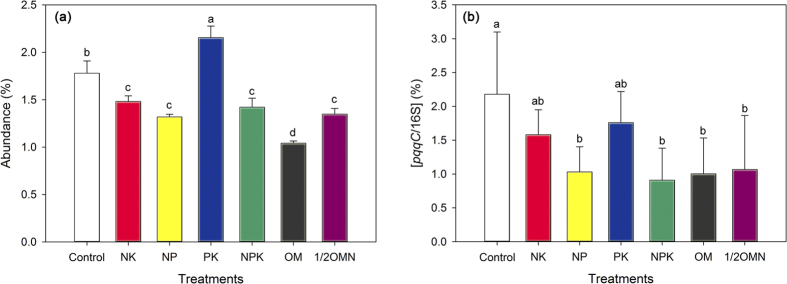
The abundance of iPSB based on 16S rRNA gene illumina sequencing data (**a**) and relative abundance of *pqqC* gene (**b**) in different fertilization treatments. Different letters within columns followed by indicate significance at *P* < 0.05.

**Figure 4:**
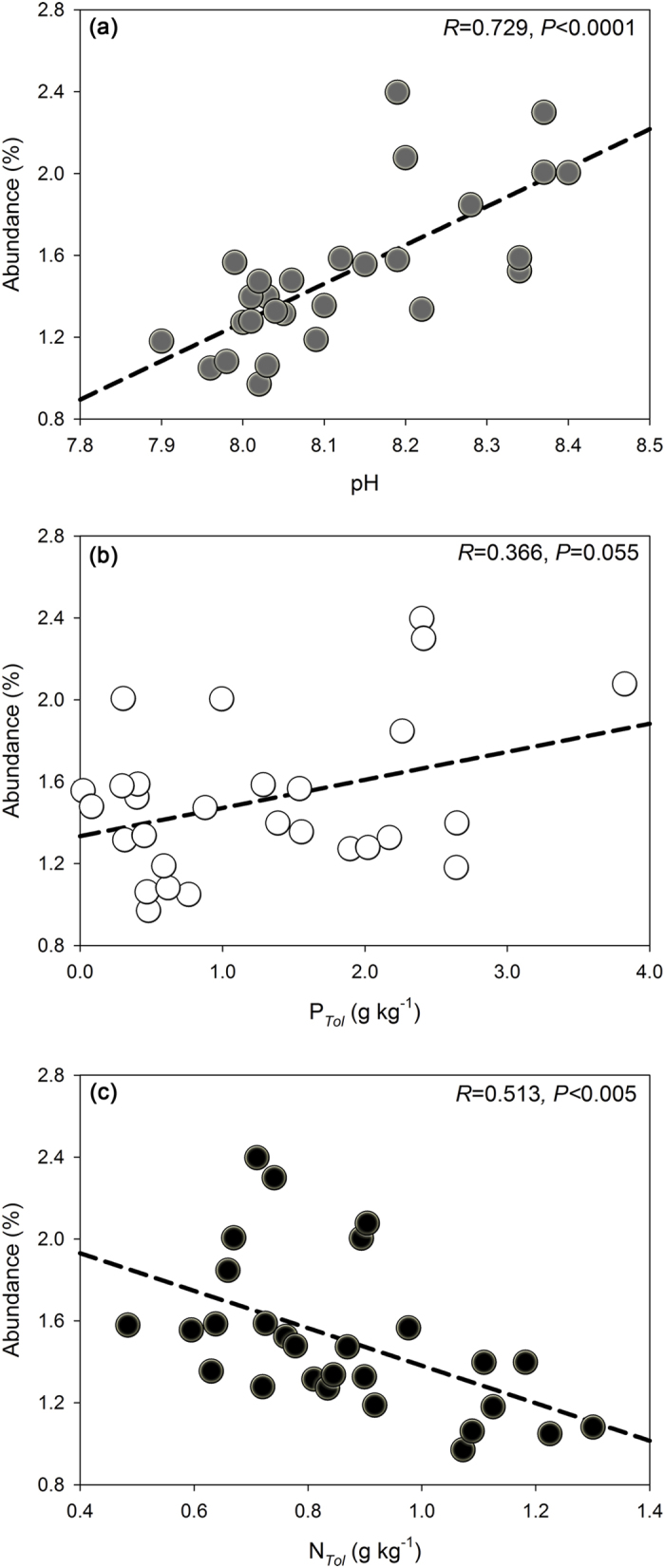
The pH (**a**), P_*Tol*_ (**b**) and N_*Tol*_ (**c**) in relation to the abundance of iPSB community. The linear regression lines are shown by dash lines. The abundance of iPSB community based on 16S rRNA gene illumina sequencing data.

Except PK treatment (without N addition), the iPSB abundance based on database alignment results in all fertilized treatments decreased significantly compared with the control treatment (Fig. 3a). Pearson’s correlation analysis showed that the iPSB abundance was strongly related to pH, C_*Tol*_, N_*Tol*_ and N_*Amo*_ (Table S6). Relative abundance of the *pqqC* gene decreased significantly (Fig. 3b). The application of *pqqC* primer showed that the control treatment was observed highest *pqqC* abundance (2.18%) while the PK treatment stood at 1.76%. With N fertilizations (NK, NP, NPK, OM and 1/2OMN treatments), [*pqqC*/16S] significantly decrease at *P* < 0.05. Linear fitting showed that iPSB abundance was but negatively correlated with N_*Tol*_ (Fig. 4c). Additionally, the content of soil biomass and the absolute abundance of 16S rRNA and *pqqC* gene were showed in Figure S6.

## Discussion

In alkaline soils, the iPSB community is an important driver for soil P mobilization. From our results (Fig. 1), iPSB composed 1~3% of the bacterial population and more than 90% of iPSB belong to Actinobacteria (*Streptomyces, Arthrobacter* and *Brevibacterium*) and Firmicutes (*Bacillus*). These findings were in accordance with previous studies showing that *Bacillus* was abundant in different types of soils^35^ and was considered to be a powerful iPSB in maize rhizosphere^36^, while *Streptomyces, Arthrobacter* and *Brevibacterium* were commonly seen as plant growth promoting bacteria (PRPB) in alkaline environments with inorganic phosphate solubilizing ability^37^,^38^,^39^. Additionally, the abundance of the iPSB community based on database alignment was pH-sensitive in this study. The abundance percentage of dominant iPSB genera increased with pH except for *Streptomyces* (Figure S7). A slight pH decline could lead to a significant decrease (*P* < 0.0001) of overall iPSB abundance (Fig. 4a), indicating that iPSB abundance is sensitive to pH perturbation. As reported previously, the increase of pH could significantly affect the distribution of bacterial communities in alkaline soil, especially increasing the relative abundance of Actinobacteria^40^. Taking together, the composition of the iPSB community in our study may be dependent on soil pH environment. Moreover, the iPSB community is indispensable for maize production in this study. Via Pearson’s correlation analysis, we found that iPSB abundance and [*pqqC*/16 S] were significantly related with maize yield (Table S6), indicating that the iPSB community may play an essential role for crop growth in alkaline soils.

Availability of soil N and P are critical for crop growth in alkaline soil. Although K was also a limiting factor for crop yield, K application had no effect on crop production or microbial parameters in alkaline soil^41^. In our study, NP and NPK showed no significant differences in soil chemical properties, the iPSB community structure and maize yield (Tables 1 and 2 and S6, Figure S8), suggesting that K is not a limiting nutrient in this soil. In contrast, compared with NK and PK treatment, NPK resulted in a significant increase in soil nutrient contents and maize yield (Table 1 and S4, Figure S8), and NK and PK were well separated from other treatments (Figure S4). Taking together, we showed that N and P are both critical limiting nutrients for crop yield in this soil.

Long-term P fertilization may have little effect on the abundance of iPSB communities based on database alignment results. In our study, soil P_*Tol*_ and P_*Osl*_ contents increased significantly in all treatments over 27 years except for the NK treatment (without P additions) (Table S4) and the PK treatment (without N additions) showed an obvious increase of iPSB abundance based on database alignment method compared with the control (Fig. 3a). The significantly higher biomass C content in NPK than NK treatment (Figure S6a) indicated that long-term P fertilization benefit for overall microbial growth in soil. Yet, we could not find any beneficial effects to increase the abundance of iPSB community (Fig. 3) and there was not significant relation between P_*Tol*_ and the iPSB abundance (Fig. 4b, *R* = 0.366, *P* = 0.055), suggesting that P-fertilization may scarcely affect the iPSB community. This was different with a previous study that compared with the NK treatment, NPK, OM and 1/2OMN enlarge the size of iPSB populations based on the plate count method^42^, which may limit to count culturable iPSB strains rather than the complete iPSB communities.

The influences of P and K inputs on iPSB communities were weaker than N additions. VPA results showed N explained substantially more variation (20.02%) than P (8.21%) and K (3.67%) to distribution of the iPSB community (Figure S5). CCA results clearly showed pH and N_*Tol*_ have more explanations than other factors (Fig. 2). The P_*Tol*_ and P_*Osl*_ values showed no significant relations with the iPSB community structure as well as K_*Tol*_ (Table 2 and Table S6). Although P fertilization may stimulate the growth of iPSB population size, the effect of N inputs seemed more crucial.

Long-term N fertilization may cause P deficiency and thus enrich the iPSB communities. Generally, N inputs promote the crop uptake of P, but long-term N fertilizations may induce relative available P deficiency for crop growth. In our study, compared with the PK treatment (without N addition), the NPK treatment showed higher maize yield but less P_*Tol*_ and P_*Osl*_ contents in soil (Table S4), suggesting that N inputs are beneficial for more P uptake by crops as reported previously^43^. The N/P ratio ([N_*Amo*_ + N_*Ntr*_]: P_*Osl*_) was often used to assess the status of soil nutrients^44^. The treatments applying with both N and P fertilizers (NP, NPK, OM and 1/2OMN) showed higher N/P ratios than the control, suggesting that P seemed relatively deficient than N after fertilizations (Table 1). That may attribute to the improved plant P uptake by long-term N inputs. Alternatively, the abundant N in soil may cause a reduction in root growth relative to shoot, leading to poorer P uptake than N^43^. According to previous studies, soil P deficiency was usually a stimulus (sugars or organic anions) for P solubilizers enrichment, including AMF and iPSB^45^. Hence, the long-term N fertilizations may stimulate the enrichment of iPSB communities.

However, in our study, long-term N inputs negatively affected the abundance of iPSB communities. In our study, N fertilization was a significant factor for the phylogenetic structure of iPSB communities (Table 3). And compared with the control, the abundance of iPSB communities of N additions (NK, NP, NPK, OM and 1/2OMN) significantly decreased based on both qPCR and database alignment results (Fig. 3). Hence we considered that long-term N additions may be detrimental for iPSB communities. According to previous studies, long-term N additions indeed showed negative effects on microbial growth^46^, and direct mechanism may be related to the toxic effect of additional ions and osmotic potentials by fertilization^47^. For iPSB communities, the modulation of their abundance may relate with P release. In this study, urea is the only N resource and could be easily degraded to be ammonium by urease which was ubiquitous in soil. The NH_4_^+^ assimilation by plant roots or microorganisms would lead to proton extrusions and pH decline, enhancing soil P release^43^. The significant decrease of soil pH by N additions (NK, NP, NPK, OM and 1/2OMN) in this study (Table 1) may contribute to more P release and relatively decrease the demands of iPSB populations.

The effect of long-term N fertilization on the abundance of iPSB community was evidently negative. Both database alignment and qPCR results showed a significant decrease of iPSB population size in N adding treatments. However, only around 400 bp fragments of 16S rRNA could be high-throughput sequencing due to the limitation of illumina sequencing technology and this short sequence length only allowed us to annotate the matched sequence as potential iPSB species since many bacteria with quite different capabilities may share the same genetic makeup in this sequencing area. Hence, a novel *pqqC* primer was designed to validate that the abundance of iPSB community was approaching identical in both methods (Fig. 3). Nevertheless, the database alignment method is reproducible and precise to track the variations of microbial communities if complete 16S rRNA sequences were provided by groundbreaking sequencing technologies in the future. At last but not least, our study here have made a breakthrough to investigate the structure of iPSB community without growth-based measurement and provide a new insight of the feedback of iPSB community to agricultural practices.

## Conclusions

Our results indicated that long-term fertilization significantly affected the phylogenetic structure of iPSB communities in an alkaline soil. The long-term N rather than P and K fertilization significantly decrease the abundance of iPSB communities based on both 16S rRNA illumina sequencing and *pqqC* gene quantification. Significant correlations were observed between iPSB abundance and soil pH and N_*Tol*_, implicated that long-term N additions may lead to soil acidification and P releases and thus modulate iPSB populations.

## Additional Information

**How to cite this article:** Zheng, B.-X. *et al*. Long-term nitrogen fertilization decreased the abundance of inorganic phosphate solubilizing bacteria in an alkaline soil. *Sci. Rep.* **7**, 42284; doi: 10.1038/srep42284 (2017).

**Publisher's note:** Springer Nature remains neutral with regard to jurisdictional claims in published maps and institutional affiliations.

## Supplementary Material

Supplementary Information

## Figures and Tables

**Table 1:** Soil chemical parameters.

Treatment	pH	C_*Tol*_ (g kg^−1^)	N_*Tol*_ (g kg^−1^)	N_*Amo*_ (mg kg^−1^)	N_*Ntr*_ (mg kg^−1^)	P_*Tol*_ (g kg^−1^)	P_*Osl*_ (mg kg^−1^)	K_*Tol*_ (g kg^−1^)	N/P
Control	8.36 ± 0.03 a	10.055 ± 0.455 bc	0.763 ± 0.046 bc	0.43 ± 0.25 c	2.63 ± 0.17 c	0.526 ± 0.314 c	7.753 ± 2.702 d	9.27 ± 1.45 a	0.43 ± 0.07 b
NK	8.11 ± 0.07 c	8.525 ± 0.185 c	0.668 ± 0.078 c	2.33 ± 0.60 a	79.54 ± 32.39 a	0.178 ± 0.149 c	8.288 ± 5.980 d	8.37 ± 0.55 a	12.03 ± 3.09 a
NP	8.02 ± 0.02 cd	11.418 ± 0.419 b	0.908 ± 0.098 b	0.69 ± 0.69 bc	17.68 ± 8.51 c	2.185 ± 0.329 ab	30.171 ± 6.029 b	10.07 ± 1.08 a	0.65 ± 0.19 b
PK	8.26 ± 0.08 b	10.378 ± 0.731 bc	0.753 ± 0.052 bc	0.93 ± 0.46 bc	3.57 ± 1.54 c	2.726 ± 0.740 a	45.860 ± 4.409 a	9.61 ± 3.45 a	0.10 ± 0.02 b
NPK	8.03 ± 0.10 cd	10.305 ± 0.707 bc	0.845 ± 0.125 bc	1.31 ± 0.98 abc	44.55 ± 26.27 b	1.756 ± 0.602 b	23.599 ± 2.298 bc	9.18 ± 2.05 a	1.99 ± 0.60 b
OM	8.00 ± 0.03 d	13.465 ± 1.109 a	1.173 ± 0.055 a	2.48 ± 0.53 a	19.16 ± 6.19 bc	0.583 ± 0.137 c	21.673 ± 4.989 c	7.62 ± 1.64 a	1.08 ± 0.23 b
1/2OMN	8.09 ± 0.10 cd	11.405 ± 0.349 b	0.935 ± 0.061 b	1.88 ± 1.50 ab	19.26 ± 6.40 bc	0.826 ± 0.416 c	23.943 ± 6.971 bc	7.21 ± 1.69 a	0.92 ± 0.15 b

Mean ± Standard Deviation (n = 4).

Different letters within rows followed by indicate significance at *P* < 0.05.

C_*Tol*_: total carbon, N_*Tol*_: total nitrogen, N_*Amo*_: ammonium nitrogen, N_*Ntr*_: nitrate nitrogen, P_*Tol*_: total phosphorus, P_*Osl*_: available phosphorus, K_*Tol*_: total potassium, N/P: the ratio of inorganic N (N_*Amo*_ + N_*Ntr*_) to P_*Osl*_.

Control: without fertilizer, NK: chemical nitrogen (N) and potassium (K) fertilizer, NP: chemical N and phosphorus (P) fertilizer, PK: chemical P and K fertilizer, NPK: chemical N, P and K fertilizer, OM: organic manures, 1/2OMN: half chemical NPK fertilizer plus half organic manure.

**Table 2:** Monte Carlo permutation tests of the impact of soil chemical properties on the overall iPSB community structure based on 16S rRNA gene sequencing data.

Variables	*R*^*2*^	*P*
pH	0.565	0.001^***^
C_*Tol*_	0.336	0.004^**^
N_*Tol*_	0.359	0.005^**^
N_*Amo*_	0.076	0.351
N_*Ntr*_	0.026	0.729
P_*Tol*_	0.149	0.128
P_*Osl*_	0.141	0.148
K_*Tol*_	0.071	0.412

*P < 0.05, ^**^P < 0.01, ^***^P < 0.001.

**Table 3:** Adonis tests of the effect of chemical fertilization on the overall iPSB community structure based on 16S rRNA gene sequences.

	*F*	*P*
Control vs NK	4.079	0.028*
Control vs NP	7.550	0.032*
Control vs PK	3.281	0.089
NPK vs PK	8.395	0.034*
NPK vs NK	0.793	0.448
NPK vs NP	0.637	0.434

*P < 0.05.
