# Supplementary material for: Long-term nitrogen fertilization decreased the abundance of inorganic phosphate solubilizing bacteria in an alkaline soil
Source: Sci Rep. 2017 Feb 9;7:42284. doi: 10.1038/srep42284 (PMC5299424; doi:10.1038/srep42284)
Supplement: Supplementary Information [file srep42284-s1.pdf]

Long-term nitrogen fertilization decreased the abundance of inorganic phosphate solubilizing bacteria in an alkaline soil

Bang-Xiao Zheng[a,b#], Xiu-Li Hao[a,c#], Kai Ding[a], Guo-Wei Zhou[a,b], Qing-Lin Chen[a,b], Jia-Bao Zhang[d*], Yong-Guan Zhu[a,e**]

[a] *Key Laboratory of Urban Environment and Health, Institute of Urban Environment, Chinese Academy of Sciences, Xiamen 361021, China*;

[b] *University of Chinese Academy of Sciences, Beijing 100049, China*;

[c] *Department of Plant and Environmental Sciences, University of Copenhagen, Frederiksberg 1871, Denmark*;

[d] *State Key Lab Soil & Sustainable Agriculture, Institute of Soil Science, Chinese Academy of Sciences, Nanjing 210008, China*;

[e] *State Key Laboratory of Urban and Regional Ecology, Research Center for Eco-Environmental Sciences, Chinese Academy of Sciences, Beijing 100085, China.*

# Bang-Xiao Zheng and Xiu-Li Hao contributed equally to this work.

* Corresponding Author

Phone: (+86) 258 6881228. Fax: (+86) 258 6881000. Email: jbzhang@issas.ac.cn

** Corresponding Author

Phone: (+86) 592 6190997. Fax: (+86) 592 6190791. E-mail: ygzhu@iue.ac.cn

Table S1. The experiment design of fertilizer application.

Treatment	Basal fertilizers									Supplementary fertilizer urea (kg N ha^{-1})
	N (kg N ha^{-1})			P (kg P_2O_5 ha^{-1})			K (kg K_2O ha^{-1})			
	Manure	Urea	Total	Manure	Super phosphate	Total	Manure	Potassium sulfate	Total	
control	0	0	0	0	0	0	0	0	0	0
NK	0	60	60	0	0	0	0	150	150	90
NP	0	60	60	0	75	75	0	0	0	90
PK	0	0	0	0	75	75	0	150	150	0
NPK	0	60	60	0	75	75	0	150	150	90
OM	150	0	150	51	24	75	65	85	150	0
1/2OMN	0	75	75	25.5	49.5	75	32.5	117.5	150	75

Table S2. Information of reference iPSB database.

No.	Accession number	Species	Strain name	Reference
1	HQ242765	*[Brevibacterium] frigoritolerans*	PSB52	1
2	HQ242742	*Acinetobacter calcoaceticus*	PSB29	1
3	KJ190162	*Acinetobacter calcoaceticus*	ICA01	2
4	KJ190163	*Acinetobacter calcoaceticus*	ICA02Ba	2
5	HQ412512	*Acinetobacter* sp.	E8.6(2011)	3
6	KC245151	*Acinetobacter* sp.	ASL12	4
7	KJ190164	*Acinetobacter* sp.	ICA03Bs	2
8	KJ190165	*Acinetobacter* sp.	ICA04Ma	2
9	JN173077	*Acinetobacter* sp.	L176(2011)	5
10	JN630808	*Advenella incenata*	PB-05	6
11	JN630809	*Advenella mimigardefordensis*	PB-06	6
12	JQ727433	*Advenella mimigardefordensis*	PB-10	6
13	KF266702	*Agrobacterium tumefaciens*	SCAUK0306	7
14	KJ748593	*Alcaligenes faecalis*	19UPMNR	8
15	KJ729608	*Alcaligenes faecalis*	6upmr	8
16	KJ748586	*Alcaligenes faecalis*	2UPMR	8
17	KJ748585	*Alcaligenes faecalis*	10UPMR	8
18	KJ748587	*Alcaligenes faecalis*	24UPMR	8
19	X80743	*Arthrobacter nicotinovorans*		9
20	HQ242763	*Arthrobacter nitroguajacolicus*	PSB50	1
21	HQ242764	*Arthrobacter pascens*	PSB51	1
22	X80740	*Arthrobacter pascens*		9
23	HQ242762	*Arthrobacter ramosus*	PSB49	1
24	X80744	*Arthrobacter ureafaciens*		9
25	HQ242766	*Bacillus aryabhattai*	PSB53	1
26	HQ242767	*Bacillus aryabhattai*	PSB54	1
27	HQ242769	*Bacillus aryabhattai*	PSB56	1
28	HQ242770	*Bacillus aryabhattai*	PSB57	1
29	HQ242771	*Bacillus aryabhattai*	PSB58	1
30	HQ242772	*Bacillus aryabhattai*	PSB59	1
31	HQ242773	*Bacillus aryabhattai*	PSB60	1
32	HQ242774	*Bacillus aryabhattai*	PSB61	1
33	HQ242775	*Bacillus aryabhattai*	PSB62	1
34	KJ729602	*Bacillus cereus*	20UPMNR	8

35	GU479999	*Bacillus circulan*s	PSSG6	[10]
36	HM637290	*Bacillus megaterium*	TSBF 723	[11]
37	HQ242768	*Bacillus megaterium*	PSB55	[1]
38	X60629	*Bacillus megaterium*		[9]
39	HM637292	*Bacillus* sp.	TSBF 739	[11]
40	KF741778	*Bacillus* sp.	ADH306	[4]
41	KF266700	*Bacillus* sp.	SCAUK0311	[7]
42	JN104596	*Bacillus* sp.	J9(2011)	[5]
43	JN104592	*Bacillus* sp.	J225(2011)	[5]
44	JN969593	*Bacillus* sp.	J255	[5]
45	JN969591	*Bacillus* sp.	L54	[5]
46	JN969592	*Bacillus* sp.	L55	[5]
47	JQ307185	*Brevundimonas diminuta*	RS1	[12]
48	JQ307186	*Brevundimonas diminuta*	RS2	[12]
49	EF113108	*Burkholderia cepacia*		[7]
50	HQ242761	*Burkholderia phytofirmans*	PSB48	[1]
51	HM637291	*Burkholderia* sp.	TSBF 699F	[11]
52	HQ412511	*Burkholderia* sp.	H5.7(2011)	[3]
53	KF761523	*Burkholderia* sp.	PSB-51	[13]
54	KF761524	*Burkholderia* sp.	PSB-69	[13]
55	KF761525	*Burkholderia* sp.	PSB-70	[13]
56	HQ242718	*Cedecea davisae*	PSB5	[1]
57	M58774	*Chryseobacterium indoltheticum*		[9]
58	AB075017	*Delftia tsuruhatensis*		[9]
59	HQ242724	*Enterobacter amnigenus*	PSB11	[1]
60	HQ242725	*Enterobacter amnigenus*	PSB12	[1]
61	HQ242726	*Enterobacter amnigenus*	PSB13	[1]
62	HQ242717	*Enterobacter asburiae*	PSB4	[1]
63	HQ242719	*Enterobacter asburiae*	PSB6	[1]
64	HQ242720	*Enterobacter asburiae*	PSB7	[1]
65	HQ242733	*Enterobacter cancerogenus*	PSB20	[1]
66	HQ242716	*Enterobacter gergoviae*	PSB3	[1]
67	HQ242714	*Enterobacter ludwigii*	PSB1	[1]
68	HQ242715	*Enterobacter ludwigii*	PSB2	[1]
69	KC245150	*Enterobacter* sp.	ADH302	[4]
70	JN104595	*Enterobacter* sp.	J157(2011)	[5]

71	JQ304269	*Enterobacter* sp.	J33a	[5]
72	JN091872	*Enterobacter* sp.	S57	[5]
73	JN104594	*Enterococcus* sp.	L177(2011)	[5]
74	JN104593	*Enterococcus* sp.	L185(2011)	[5]
75	JN173076	*Enterococcus* sp.	L191(2011)	[5]
76	KC245152	*Escherichia* sp.	ASG34	[4]
77	Y18310	*Gordonia polyisoprenivorans*		[9]
78	HQ242727	*Klebsiella oxytoca*	PSB14	[1]
79	KF761520	*Klebsiella* sp.	PSB-08	[13]
80	KF741779	*Kurthia* sp.	ASG16	[4]
81	HQ242721	*Leclercia adecarboxylata*	PSB8	[1]
82	HQ242722	*Leclercia adecarboxylata*	PSB9	[1]
83	HQ242723	*Leclercia adecarboxylata*	PSB10	[1]
84	KF266699	*Mesorhizobium* sp.	SCAUK0307	[7]
85	KF741777	*Paenibacillus massiliensis*	ASG41	[4]
86	KF741780	*Paenibacillus panacisoli*	ATZ304	[4]
87	HM776636	*Paenibacillus* sp.	TSBF 828	[11]
88	HM776635	*Paenibacillus* sp.	TSBF 845	[11]
89	KF741776	*Paenibacillus* sp.	ASG64	[4]
90	HQ242739	*Pantoea agglomerans*	PSB26	[1]
91	HQ242740	*Pantoea agglomerans*	PSB27	[1]
92	HQ242741	*Pantoea agglomerans*	PSB28	[1]
93	HQ242738	*Pantoea conspicua*	PSB25	[1]
94	D12789	*Phyllobacterium myrsinacearum*	IAM 13584	[9]
95	HQ242755	*Pseudomonas brassicacearum*	PSB42	[1]
96	HQ242748	*Pseudomonas corrugata*	PSB35	[1]
97	HQ242749	*Pseudomonas frederiksbergensis*	PSB36	[1]
98	HQ242750	*Pseudomonas frederiksbergensis*	PSB37	[1]
99	HQ242746	*Pseudomonas koreensis*	PSB33	[1]
100	HQ242753	*Pseudomonas lini*	PSB40	[1]
101	HQ242754	*Pseudomonas lini*	PSB41	[1]
102	HQ242757	*Pseudomonas lini*	PSB44	[1]
103	HQ242751	*Pseudomonas mandelii*	PSB38	[1]
104	HQ242752	*Pseudomonas mandelii*	PSB39	[1]
105	HQ242756	*Pseudomonas mediterranea*	PSB43	[1]
106	HQ242758	*Pseudomonas mediterranea*	PSB45	[1]

107	HQ242759	*Pseudomonas mediterranea*	PSB46	[1]
108	HQ242760	*Pseudomonas mediterranea*	PSB47	[1]
109	HQ242745	*Pseudomonas moraviensis*	PSB32	[1]
110	HQ242747	*Pseudomonas moraviensis*	PSB34	[1]
111	HQ242743	*Pseudomonas putida*	PSB30	[1]
112	HQ242744	*Pseudomonas putida*	PSB31	[1]
113	NR_029063	*Pseudomonas rhizosphaerae*	IH5	[14]
114	HQ412501	*Pseudomonas* sp.	A10.1(2011)	[3]
115	HQ412502	*Pseudomonas* sp.	D5.2(2011)	[3]
116	HQ412503	*Pseudomonas* sp.	D7.2(2011)	[3]
117	HQ412504	*Pseudomonas* sp.	E2.2(2011)	[3]
118	HQ412505	*Pseudomonas* sp.	E3.6(2011)	[3]
119	HQ412506	*Pseudomonas* sp.	E8.5(2011)	[3]
120	HQ412507	*Pseudomonas* sp.	E8.7(2011)	[3]
121	HQ412508	*Pseudomonas* sp.	F4.2(2011)	[3]
122	HQ412509	*Pseudomonas* sp.	F4.6(2011)	[3]
123	HQ412510	*Pseudomonas* sp.	G10.5(2011)	[3]
124	KF266705	*Pseudomonas* sp.	SCAUK0301	[7]
125	KF266706	*Pseudomonas* sp.	SCAUK0309	[7]
126	HQ242729	*Raoultella ornithinolytica*	PSB16	[1]
127	HQ242730	*Raoultella ornithinolytica*	PSB17	[1]
128	HQ242731	*Raoultella ornithinolytica*	PSB18	[1]
129	HQ242732	*Raoultella ornithinolytica*	PSB19	[1]
130	HQ242728	*Raoultella terrigena*	PSB15	[1]
131	KF741781	*Rhizobium* sp.	AX7	[4]
132	KF266696	*Rhizobium* sp.	SCAUK0315	[7]
133	KF266701	*Rhizobium* sp.	SCAUK0313	[7]
134	KF266697	*Rhizobium* sp.	SCAUK0345	[7]
135	KF266698	*Rhizobium* sp.	SCAUK0353	[7]
136	X80618	*Rhodococcus erythropolis*	DSM43188T	[9]
137	HQ242737	*Serratia grimesii*	PSB24	[1]
138	HQ242736	*Serratia marcescens*	PSB23	[1]
139	AJ233431	*Serratia marcescens*	DSM 30121	[9]
140	KJ729609	*Serratia plymuthica*	31upmr	[8]
141	JN091871	*Serratia* sp.	J21	[5]
142	JN091870	*Serratia* sp.	J145	[5]

143	JN969594	*Serratia* sp.	J260	5
144	JN173078	*Serratia* sp.	S93(2011)	5
145	GQ165511	*Serratia* sp.	S119(2009)	5
146	HQ242734	*Serratia ureilytica*	PSB21	1
147	HQ242735	*Serratia ureilytica*	PSB22	1
148	KF741782	*Shigella* sp.	ASG33	4
149	KF761521	*Staphylococcus* sp.	PSB-18	13
150	KF761522	*Staphylococcus* sp.	PSB-21	13
151	KF266703	*Streptomyces* sp.	SCAUK0310	7
152	KF266704	*Streptomyces* sp.	SCAUK0356	7
153	KJ783452	*Vagococcus carniphilus*	17upmnr	8
154	GQ165512		J49	5

Table S3. Amplification of *pqqC* sequences from isolated iPSB strains.

Strains	Accession number	Amplicon[*]	Pi solubilizing ability[†]
Bacillus megaterium 01-A3	KU647195	-	C
Bacillus megaterium 02-A7	KU647196	-	C
Pseudomonas frederiksbergensis 03-D2	KU647197	+	B
Rhodococcus opacus 04-OD7	KU647198	+	A
Arthrobacter phenanthrenivorans 05-OD11	KU647199	+	A
Arthrobacter defluvii 06-OD12	KU647200	-	B
Arthrobacter chlorophenolicus 07-OD13	KU647201	+	A
Arthrobacter oxydans 08-OY2	KU647202	-	A
Arthrobacter sp. 09-OY5	KU647203	+	B
Bacillus megaterium 10-Y11	KU647204	-	C
Pseudomonas frederiksbergensis 11-D3	KU647205	+	C
Massilia putida 12-OD1	KU647206	+	C
Duganella sp. 13-D4	KU647207	+	A
Bacillus megaterium 14-Y2	KU647208	-	C
Pseudoduganella sp. 15-Y6	KU647209	+	B
Bacillus megaterium 16-Y9	KU647210	-	C
Bacillus megaterium 17-Y5	KU647211	-	C
Variovorax paradoxus 19-D4	KU647212	+	B
Rhizobium leguminosarum 20-OD2	KU647213	+	A
Rhodanobacter sp. 21-Y7	KU647214	+	A
Bacillus megaterium 22-A1	KU647215	-	C
Pseudomonas frederiksbergensis 23-D2	KU647216	+	B
Bacillus megaterium 24-Y916	KU647217	-	C
Rhodanobacter sp. 25-Y8	KU647218	+	A
Bacillus megaterium 26-Y91	KU647219	-	B
Bacillus megaterium 27-Y93	KU647220	-	C
Bacillus megaterium 28-Y911	KU647221	-	D
Bacillus megaterium 29-Y924	KU647222	-	D
Bacillus megaterium 30-Y1411	KU647223	-	D
Bacillus megaterium 31-Y142	KU647224	-	C
Arthrobacter sp. 32-OD9	KU647225	+	B
Streptomyces tumescens 33-X1	KU647226	+	A
Streptomyces prasinopilosus 34-Y1	KU647227	+	A
Streptomyces rishiriensis 35-Y3	KU647228	+	B

Kurthia zopfii 36-Y7	KU647229	+	C
Rhodanobacter sp. 37-Y8	KU647230	+	A
Bacillus megaterium 38-Y92	KU647231	-	C
Bacillus megaterium 39-Y94	KU647232	-	C
Bacillus megaterium 40-Y95	KU647233	-	D
Bacillus megaterium 41-Y99	KU647234	-	D
Bacillus megaterium 42-Y910	KU647235	-	B
Bacillus megaterium 43-Y912	KU647236	-	B
Bacillus megaterium 44-Y913	KU647237	-	B
Bacillus megaterium 45-Y914	KU647238	-	C
Bacillus megaterium 46-Y923	KU647239	-	C
Bacillus megaterium 47-Y141	KU647240	-	B
Rhizobium sp. 48-Y930	KU647241	+	A
Bacillus megaterium 49-Y1412	KU647242	-	D
Rhizobium sp. 50-Y1414	KU647243	+	A
Burkholderia cepacia 51-Y1415	KU647244	+	A
Arthrobacter defluvii 52-OD12	KU647245	+	B
Bacillus acidiceler 53-Q11	KU647246	-	D
Streptomyces prasinopilosus 54-Y1	KU647247	+	B
Pseudomonas frederiksbergensis 55-D3	KU647248	+	A
Burkholderia phytofirmans 56-OY3	KU647249	+	A
Variovorax paradoxus 57-Y925	KU647250	+	A
Telluria mixta 58-Y97	KU647251	+	C
Sphingomonas koreensis 59-Y96	KU647252	-	A
Streptomyces flaveolus 60-OD3	KU647253	+	A
Rhodanobacter sp. 61-Y8	KU647254	+	B
Streptomyces sp. 62-Y930	KU647255	+	A
Rhodococcus cercidiphylli 63-OD5	KU647256	+	A
Bacillus megaterium 64-Y98	KU647257	-	C
Bacillus megaterium 65-Y918	KU647258	-	B
Bacillus megaterium 66-Y143	KU647259	-	C
Rhodococcus sp. 67-OD10	KU647260	+	B
Arthrobacter oxydans 68-OY1	KU647261	+	A
Pseudomonas sp. 69-Y94	KU647262	+	B
Bacillus megaterium 70-Y917	KU647263	-	B
Pseudomonas sp. 71-Y928	KU647264	+	A

Bacillus megaterium 72-Y13	KU647265	-	C
Bacillus megaterium 73-Y142	KU647266	-	C
Streptomyces sp. 74-Y144	KU647267	+	A
Leifsonia shinshuensis 75-Y145	KU647268	+	A
Bacillus megaterium 76-Y149	KU647269	-	B
Streptomyces sp. 77-Y1410	KU647270	+	A
ǂ *Escherichia Coli* BL21		-	
ǂ *Paracoccus denitrificans*		-	
ǂ *Mycobacterium rufum*		-	

* The positive amplicon was marked with '+', otherwise '-'.

† The ability of Pi solubilizing was described in materials and methods. The capital letter represents the range: A, < 40 mg L^{-1}; B, 40-80 mg L^{-1}; C, 80-120 mg L^{-1}; D, > 120 mg L^{-1}.

ǂ *Escherichia Coli* BL21, *Paracoccus denitrificans* and *Mycobacterium rufum* were used as negative control strain, which was tested without inorganic P solubilization ability.

Table S4. Total phosphorus, available phosphorus and maize yield.

Treatment	P_{Tol} (g kg^{-1})			P_{Osl} (mg kg^{-1})			Yield (kg ha^{-1})		
Year	1994*	2007†	2015	1994*	2007†	2015	1993*	2008‡	2015
control	0.486	0.540	0.526±0.314 c	1.230	1.800	7.753±2.702 d	1427	1003	1628±30.46 f
NK	0.492	0.550	0.178±0.149 c	1.310	2.400	8.288±5.980 d	1514	958	1828±130.93 f
NP	0.544	0.740	2.185±0.329 ab	2.900	14.300	30.171±6.029 b	8717	7418	10010±370.91 d
PK	0.576	0.850	2.726±0.740 a	10.300	30.500	45.860±4.409 a	1745	1880	2961±267.01 e
NPK	0.547	0.710	1.756±0.602 b	3.170	14.300	23.599±2.298 bc	8502	8892	11355±142.53 c
OM	0.544	0.650	0.583±0.137 c	6.210	25.100	21.673±4.989 c	7053	-	12310±71.38 a
1/2OMN	0.534	0.710	0.826±0.416 c	3.810	22.200	23.943±6.971 bc	8849	-	11786±354.00 b

Mean±Standard Deviation (n=4).

Different letters within rows followed by indicate significance at $P < 0.05$.

*The data was referred from ref [15].

†The data was referred from ref [16].

‡The data was referred from ref [17].

Table S5 Counts of aligned OTU (> 99% similarities) that were identified as iPSB at species level.

Aligned iPSB species	CK				NK				NP				PK				NPK				OM				1/2OMN			
	1	2	3	4	1	2	3	4	1	2	3	4	1	2	3	4	1	2	3	4	1	2	3	4	1	2	3	4
Acinetobacter sp. (JN173077)	0	0	0	0	0	0	2	0	1	0	2	0	4	2	0	0	1	0	0	0	0	0	2	0	1	0	0	0
Alcaligenes faecalis (KJ748587)	0	0	0	0	0	0	0	0	0	0	1	0	0	0	0	0	0	0	0	0	0	0	0	0	0	0	0	0
Arthrobacter nicotinovorans (X80743)	0	0	1	0	0	0	0	1	0	1	3	1	1	0	0	0	0	0	1	0	2	2	0	1	2	0	2	2
Arthrobacter pascens (X80740)	314	908	635	1121	698	851	676	902	542	374	491	322	927	1294	854	881	168	616	471	637	284	242	405	299	393	529	421	559
Arthrobacter ramosus (HQ242762)	5	8	2	4	6	10	5	9	28	18	16	7	37	29	5	8	7	20	8	14	11	15	8	3	12	10	6	8
Arthrobacter ureafaciens (X80744)	0	0	0	0	0	0	0	0	0	0	0	0	0	0	0	0	0	0	0	0	0	0	0	0	0	1	0	0
Bacillus cereus (KJ729602)	130	235	136	166	116	111	69	120	100	88	119	49	234	260	149	154	43	75	55	58	88	70	62	81	105	106	57	97
Bacillus sp. (JN104596)	15	47	21	23	34	41	23	30	15	10	11	7	19	19	12	10	9	16	5	10	13	10	11	17	19	0	9	18
Bacillus sp. (JN969591)	0	3	0	0	1	0	1	2	1	0	1	0	0	2	1	2	1	0	0	0	1	0	0	2	1	1	0	0
Bacillus sp. (JN969592)	1	0	0	0	1	3	0	0	1	0	1	0	2	3	2	0	0	0	0	0	0	1	0	0	3	0	0	1
Bacillus sp. (JN969593)	120	307	154	216	109	133	69	106	91	75	58	41	190	208	144	158	33	65	42	49	35	54	55	63	72	1	41	70
Brevibacterium frigoritolerans (HQ242765)	92	231	122	151	130	136	90	127	97	77	95	35	113	129	75	83	68	90	55	50	129	87	96	96	171	159	64	106
Burkholderia phytofirmans (HQ242761)	0	0	1	0	0	0	0	0	0	0	0	0	0	0	0	0	0	0	1	0	0	0	0	0	0	0	0	0
Burkholderia sp. (KF761523)	0	0	1	0	0	0	0	0	0	0	0	0	0	0	0	0	0	0	0	0	0	0	0	0	0	0	0	0
Delftia tsuruhatensis (AB075017)	2	2	0	0	2	1	0	1	2	1	1	0	1	1	2	0	0	0	0	0	0	0	0	1	1	1	1	0
Enterobacter sp. (JN091872)	1	0	0	1	0	0	3	3	0	1	3	1	2	1	0	2	2	2	1	0	1	1	0	0	2	10	1	0
Enterobacter sp. (JQ304269)	0	0	0	0	0	0	0	0	0	0	0	0	0	0	0	0	0	1	0	0	0	0	0	0	0	73	0	0
Enterococcus sp. (JN173076)	0	0	0	1	1	0	0	0	0	0	0	0	0	0	0	0	0	0	0	0	1	1	0	0	0	18	0	0
Gordonia polyisoprenivorans (Y18310)	0	0	0	0	0	0	0	0	0	0	0	0	0	1	0	0	0	0	0	0	0	0	1	0	0	0	0	0
Klebsiella oxytoca (HQ242727)	0	0	0	0	0	0	0	0	0	0	1	0	0	0	0	0	0	0	0	0	0	0	0	0	0	0	0	0
Klebsiella sp. (KF761520)	0	0	0	0	0	0	0	0	0	0	0	0	1	0	0	0	0	0	0	0	0	0	0	0	0	1	0	0
Kurthia sp. (KF741779)	0	0	0	0	0	0	0	0	0	0	0	0	0	0	0	0	0	0	0	0	0	0	1	0	1	0	1	1
Mesorhizobium sp. (KF266699)	0	0	0	0	0	0	0	0	0	0	0	0	0	0	0	0	0	0	0	0	0	0	1	0	0	0	0	0
Paenibacillus panacisoli (KF741780)	0	1	0	5	2	2	2	3	1	0	0	0	0	0	2	0	0	0	0	0	0	0	0	0	1	0	0	1
Paenibacillus sp. (KF741776)	0	5	4	0	1	5	1	3	0	0	1	1	1	0	3	0	2	1	0	1	3	1	0	1	0	1	0	0
Paenibacillus sp. (KF741776)	0	0	0	0	0	0	0	0	0	0	0	0	0	0	0	0	0	0	0	0	0	0	0	0	2	0	0	1
Pantoea agglomerans (HQ242740)	2	4	6	5	4	4	7	0	12	3	3	1	6	10	14	5	6	4	1	1	9	5	4	1	8	5	9	0
Phyllobacterium myrsinacearum (D12789)	21	13	21	18	18	19	22	19	38	18	26	13	13	9	22	11	12	29	21	37	23	24	31	34	46	31	18	29
Pseudomonas mandelii (HQ242752)	1	4	2	3	1	3	0	1	2	4	2	4	5	18	1	5	3	4	3	2	6	2	2	3	6	6	2	6
Pseudomonas mediterranea (HQ242760)	0	0	1	1	0	0	0	1	1	1	0	1	0	0	1	0	1	1	1	0	1	0	0	0	3	1	1	0
Pseudomonas putida (HQ242744)	4	9	3	1	4	14	3	4	7	4	4	1	2	4	1	3	0	3	5	2	11	3	10	3	243	8	5	3
Pseudomonas rhizosphaerae (NR_029063)	0	0	0	0	0	0	0	0	0	0	1	0	0	0	0	0	1	0	0	0	0	0	0	0	0	0	0	0
Pseudomonas sp. (HQ412509)	0	0	0	0	0	0	0	0	0	0	0	0	0	0	0	0	0	0	0	0	0	0	0	0	4	0	0	0
Pseudomonas sp. (KF266705)	2	0	0	1	7	0	1	1	4	1	1	1	1	1	2	1	0	1	2	0	2	1	2	2	45	46	0	2
Pseudomonas sp. (KF266706)	0	0	0	0	0	0	0	0	0	1	1	0	2	0	1	0	0	0	0	0	0	0	0	0	0	1	0	0
Rhizobium sp. (KF266698)	1	2	0	3	1	2	2	2	0	2	4	1	3	8	4	1	0	6	1	6	1	11	2	3	1	0	2	3
Rhodococcus erythropolis (X80618)	1	3	0	0	3	3	1	0	4	1	8	0	1	1	1	2	0	2	0	2	3	1	1	1	1	8	1	3
Serratia marcescens (AJ233431)	0	0	0	0	0	0	0	0	0	0	0	0	0	0	1	0	0	0	0	0	0	0	0	0	0	0	0	0
Serratia ureilytica (HQ242735)	0	0	1	0	0	0	1	0	0	1	0	0	0	0	8	0	0	0	0	0	0	1	0	0	0	0	1	0
Shigella sp. (KF741782)	0	0	0	0	0	0	0	0	0	0	0	0	0	0	0	0	0	0	0	0	0	0	0	0	1	3	0	0
Streptomyces sp. (KF266703)	12	22	20	17	19	24	17	22	37	40	34	31	14	21	23	8	8	28	24	43	24	20	45	42	31	5	52	69
Streptomyces sp. (KF266704)	26	50	47	55	28	58	37	59	49	50	93	37	36	54	54	46	25	58	50	54	51	47	47	59	85	53	64	184
Total	750	1854	1178	1792	1186	1420	1032	1416	1033	771	981	554	1615	2075	1382	1380	390	1022	747	966	699	599	786	712	1260	1078	758	1163

Table S6. Pearson's correlation between soil chemical and biological properties.

	[*pqqC*/16S]	iPSB abundance	Maize yield
pH	**0.477** *	**0.729** **	**-0.743** **
C_{Tol}	-0.101	**-0.451** *	**0.588** **
N_{Tol}	-0.199	**-0.513** **	**0.606** **
N_{Amo}	-0.137	**-0.476** *	0.220
N_{Ntr}	-0.074	-0.296	-0.082
P_{Tol}	-0.050	0.366	0.094
P_{Osl}	-0.166	0.308	0.212
K_{Tol}	0.151	0.209	-0.191
[*pqqC*/16S]		0.334	**-0.600** **
iPSB abundance			**-0.682** **

$^{*}P < 0.05$, $^{**}P < 0.01$.

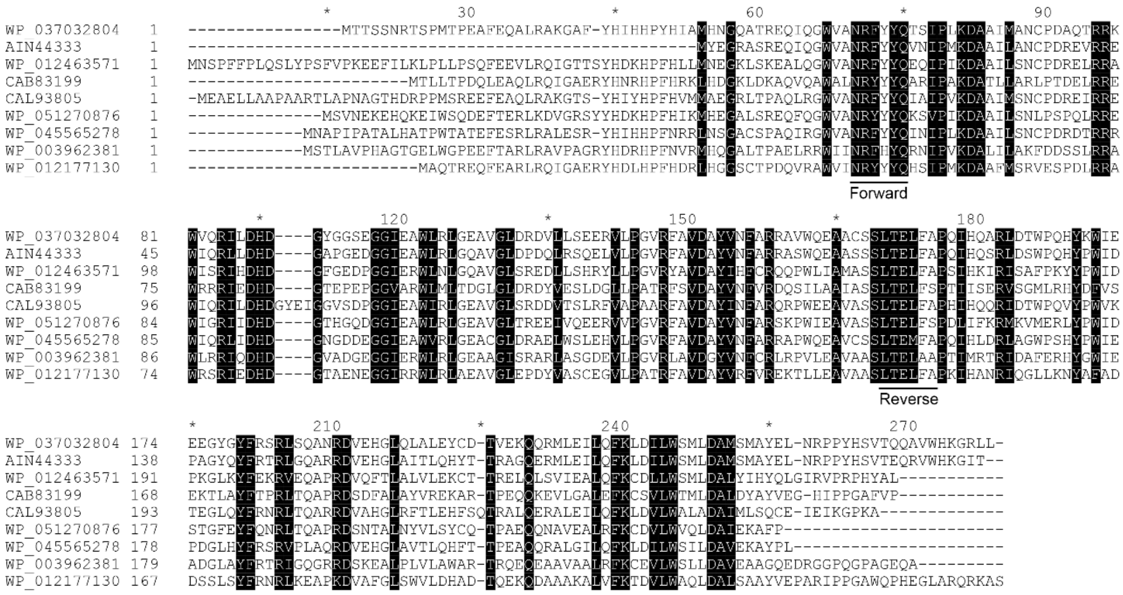

Figure S1

Alignment of pqqC amino acid sequences among different species. Perfect matches are boxed with a black background. The underlined sequences indicated the location of primers designed. WP_037032804, pyrroloquinoline quinone biosynthesis protein C from *Rahnella* sp. (Gammaproteobacteria); AIN44333, PqqC from *Pseudomonas fluorescens* (Gammaproteobacteria); WP_012463571, pyrroloquinoline quinone biosynthesis protein C from *Methylacidiphilum infernorum* (Verrucomicrobia); CAB83199, PqqC protein from *Gluconobacter oxydans* (Alphaproteobacteria); CAL93805, coenzyme PQQ synthesis protein C from *Azoarcus* sp. BH72 (Betaproteobacteria); WP_051270876, pyrroloquinoline quinone biosynthesis protein C from *Bacillus* sp. URHB0009 (Firmicutes); WP_0455652, pyrroloquinoline quinone biosynthesis protein C from *Burkholderia ubonensis* (Betaproteobacteria); WP_0039623, pyrroloquinoline quinone biosynthesis protein PqqC from *Streptomyces clavuligerus* (Actinobacteria); WP_0121771, pyrroloquinoline quinone biosynthesis protein C from *Dinoroseobacter shibae* (Alphaproteobacteria).

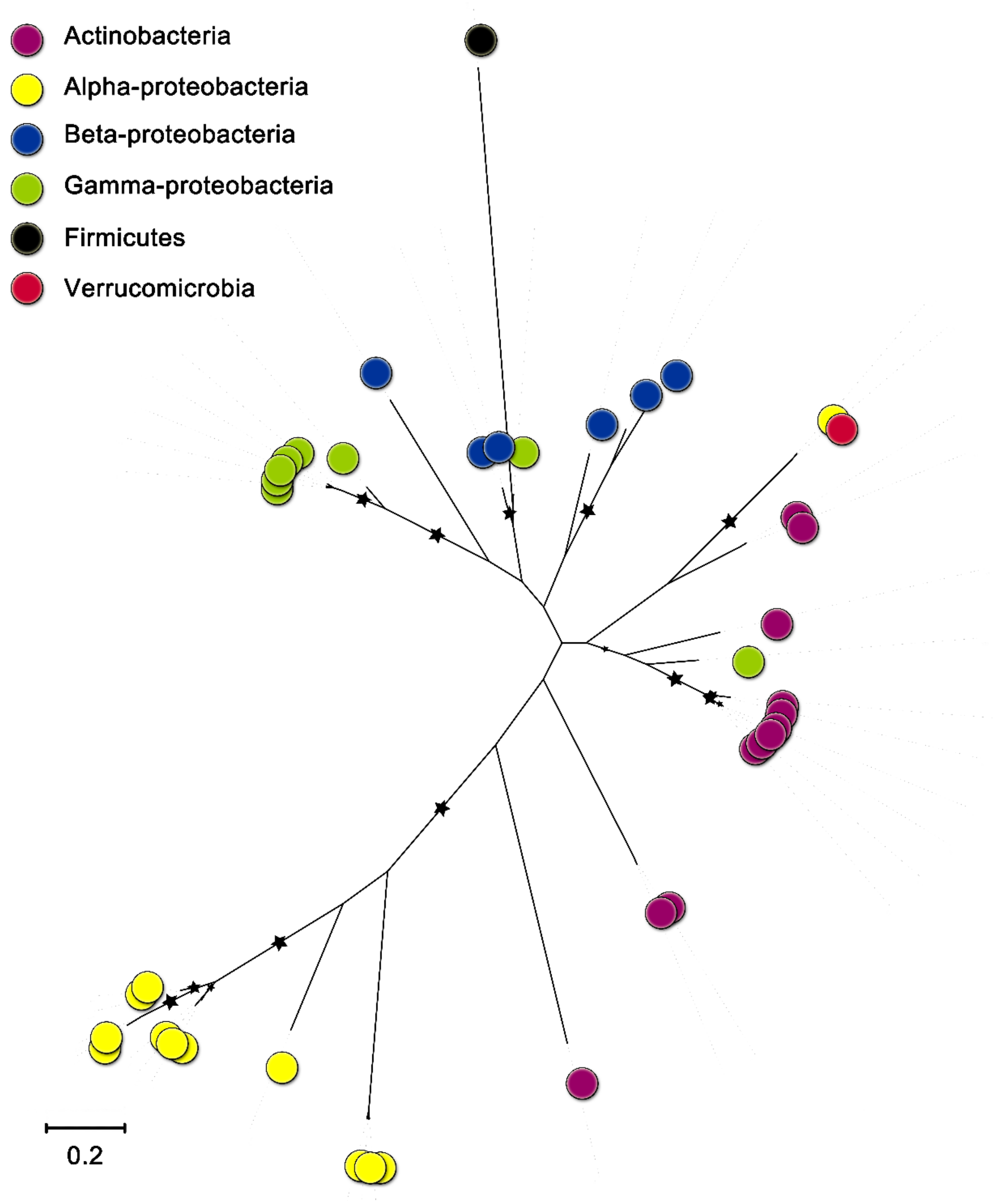

Figure S2

Unrooted maximum likelihood phylogeny of *pqqC* amino-acid sequences. Bootstrap values (500 replicates) >50% are denoted by asteriform above the branches. The branch lengths corresponding to sequence differences are indicated by the scale bar.

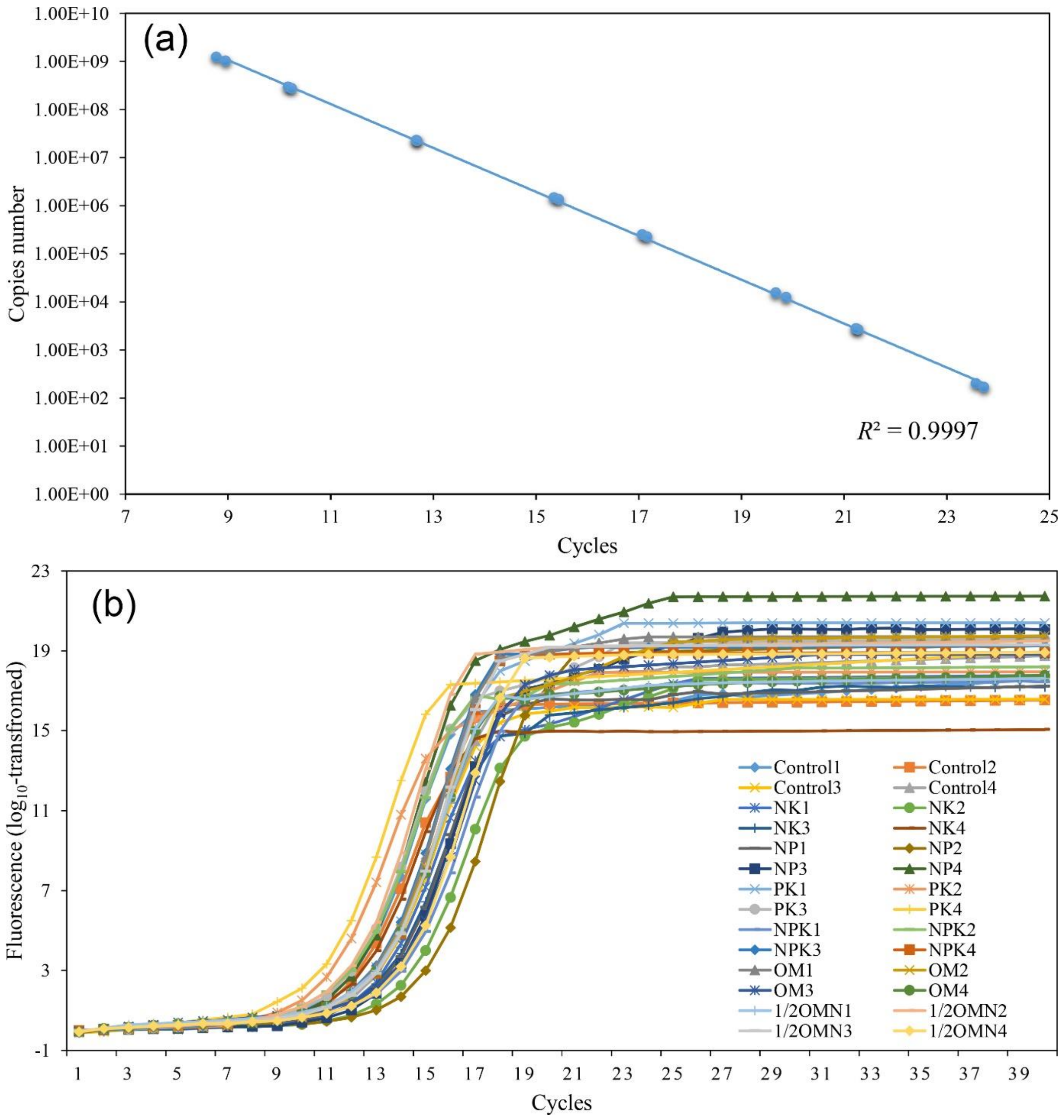

Figure S3

The standard curve (a) and log-transformed fluorescence curves (b) of *pqqC* gene amplification.

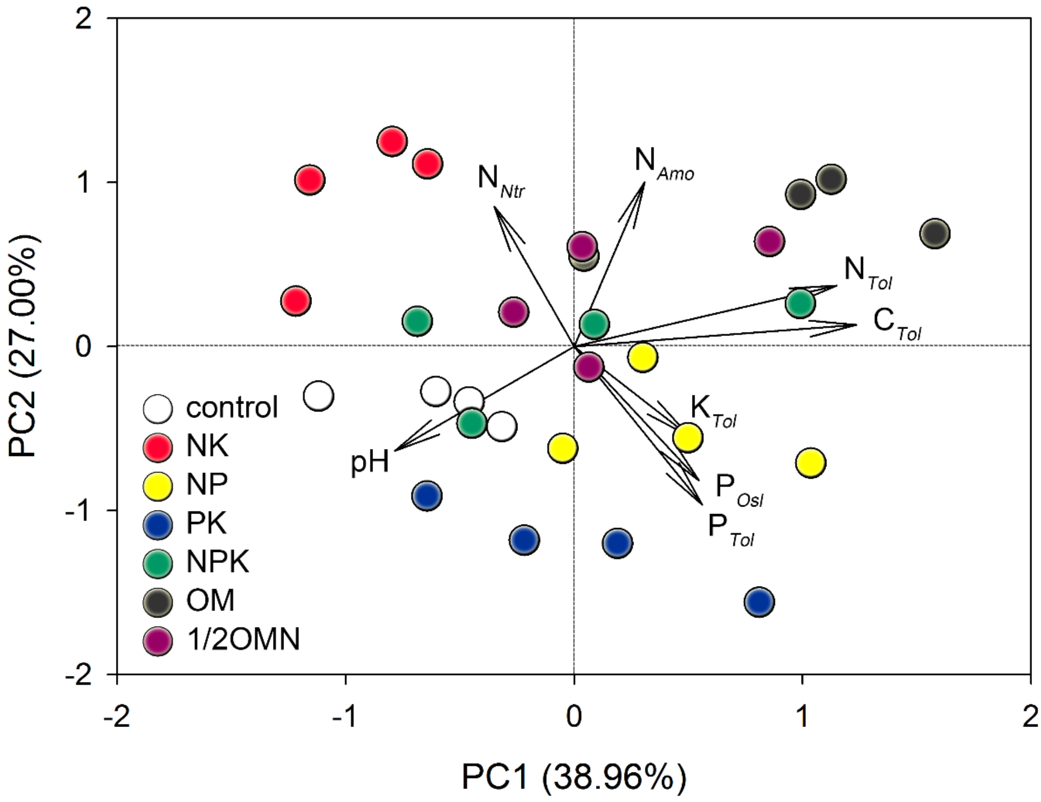

Figure S4 Principal component analysis (PCA) of soil chemical properties.

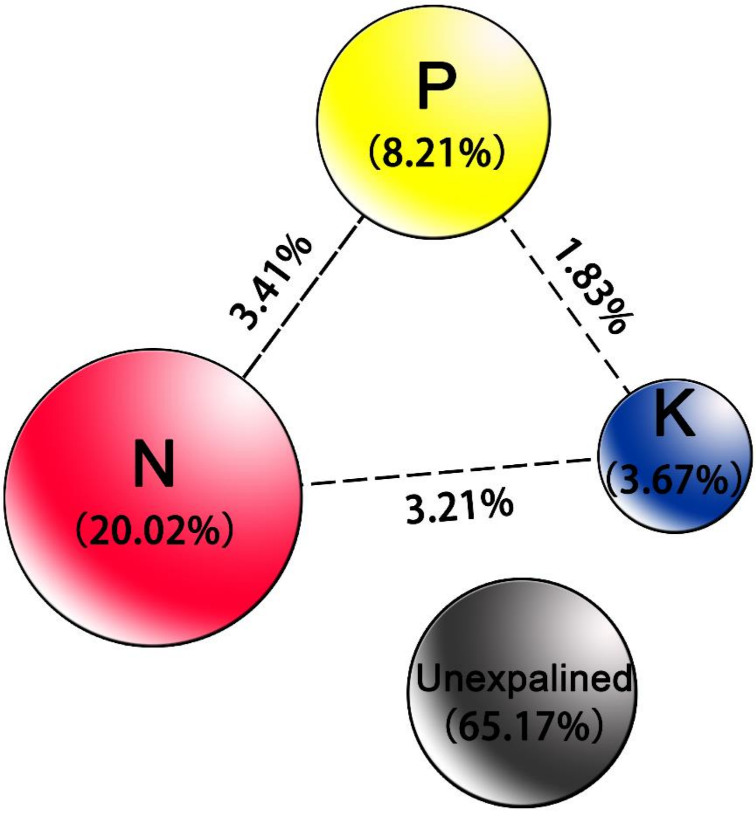

Figure S5

Variation portioning analysis (VPA) of iPSB community structure explained by soil chemical properties based on 16S rRNA sequencing data. Environmental variables include N (N_{Tol}, N_{Amo}, and N_{Ntr}), P (P_{Tol} and P_{Osl}) and K (K_{Tol}).

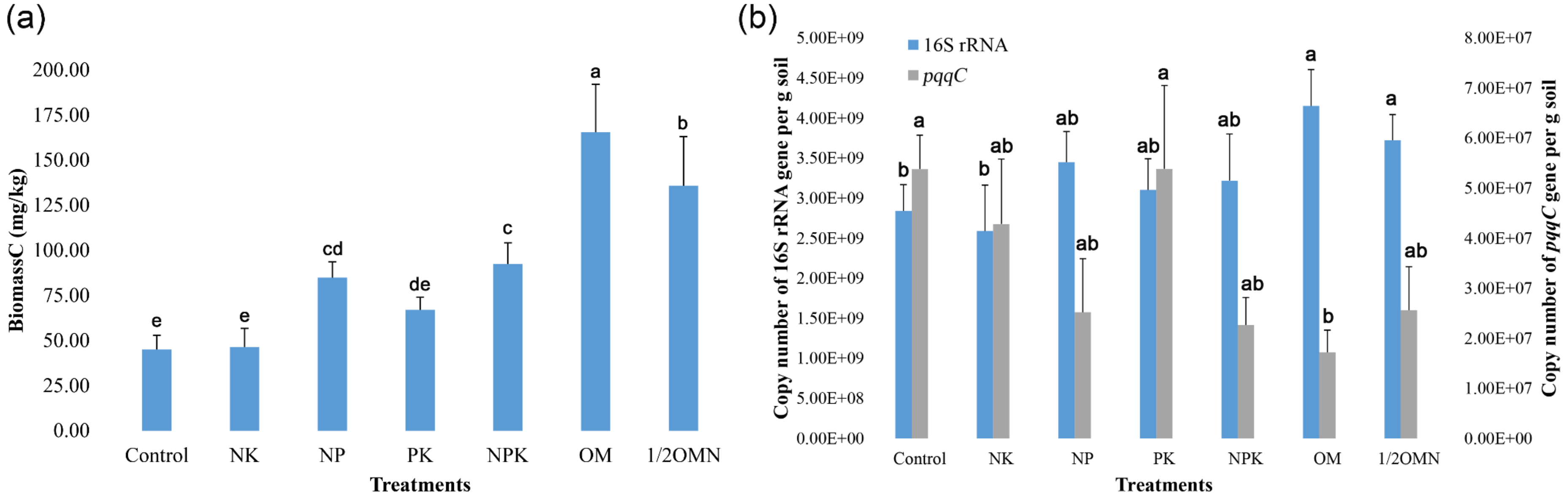

Figure S6

The biomass C (a), absolute copy number of 16S rRNA and *pqqC* gene based on soil gram (b) in different treatments. Different letters within columns followed by indicate significance at $P < 0.05$.

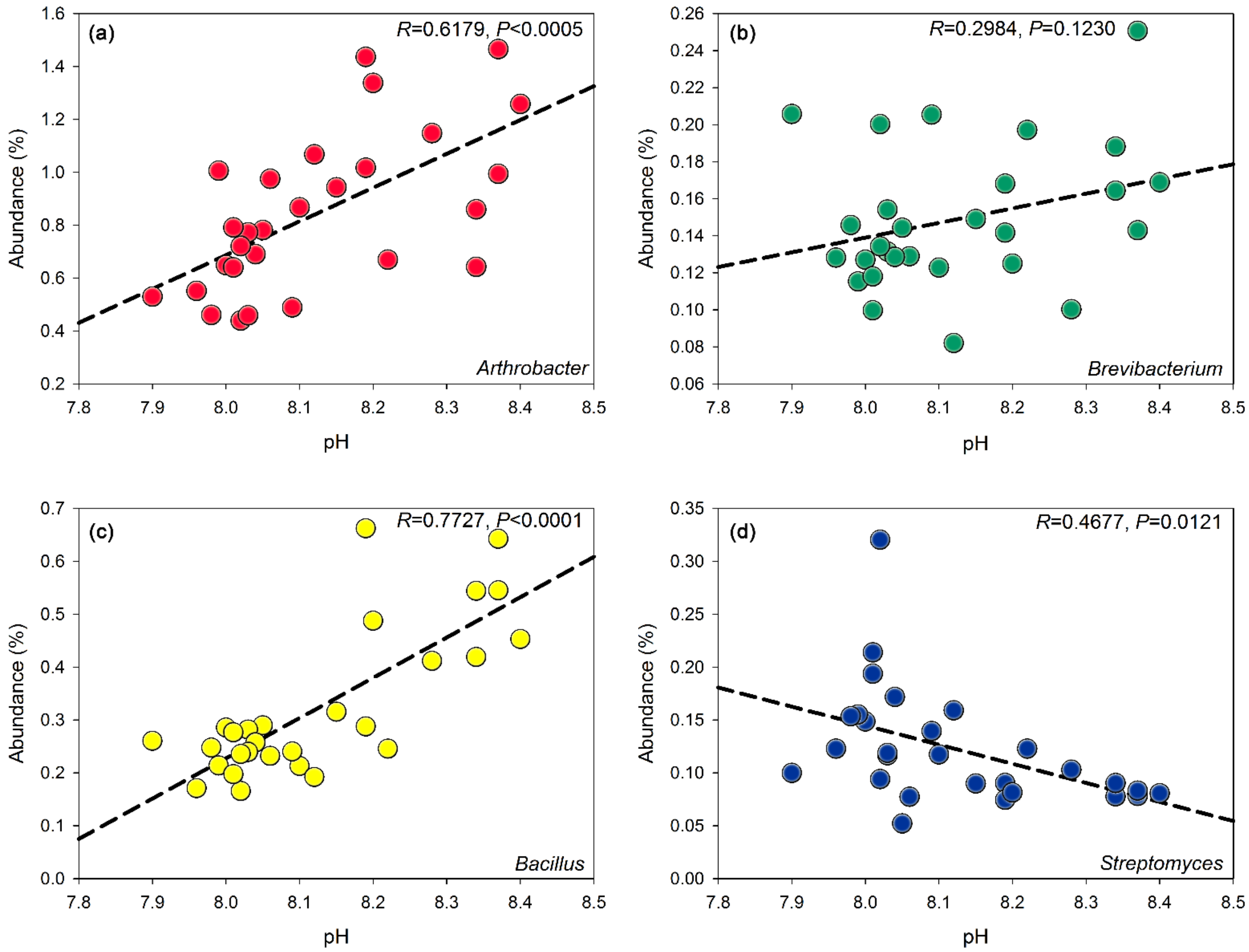

Figure S7

The effect of pH on relative abundance of dominant iPSB genera.

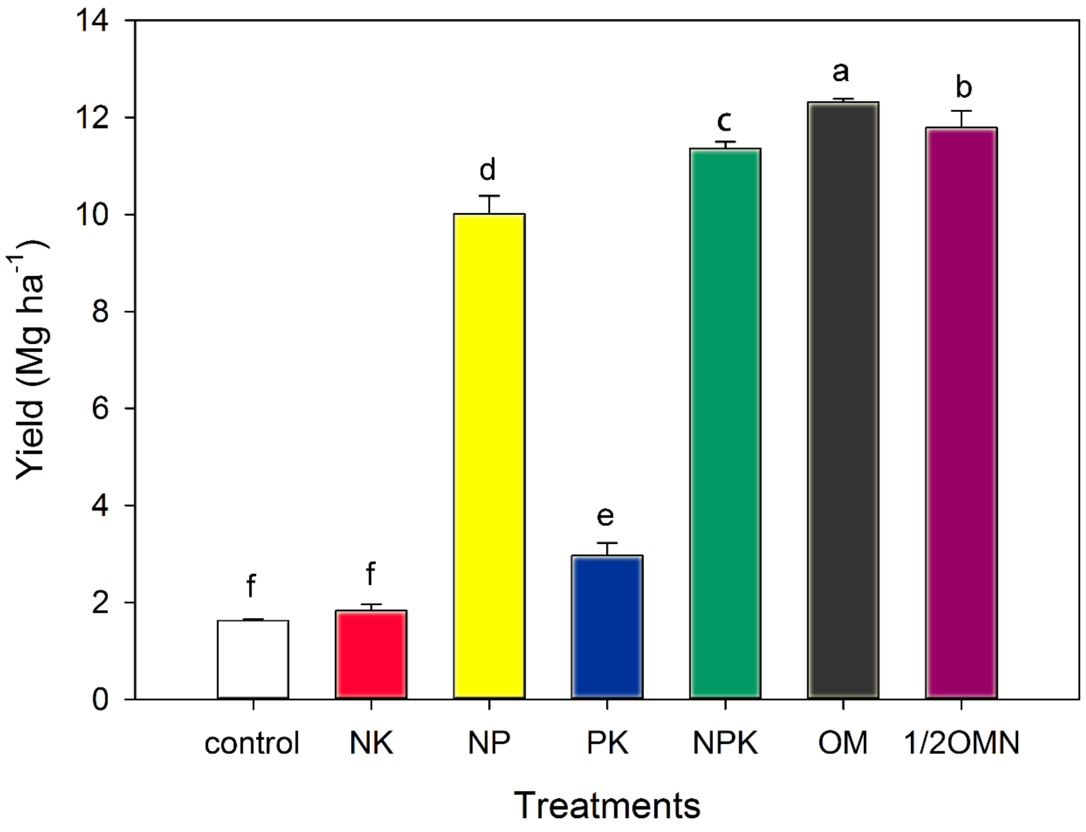

Figure S8

The maize yield in different fertilization treatments. Different letters within columns followed by indicate significance at $P < 0.05$.

References

1 Yang, P.-X. *et al.* Phosphate Solubilizing Ability and Phylogenetic Diversity of Bacteria from P-Rich Soils Around Dianchi Lake Drainage Area of China. *Pedosphere* **22**, 707–716 (2012).

2 Moreno-Ramirez, L., Gonzalez-Mendoza, D., Cecena-Duran, C. & Grimaldo-Juarez, O. Molecular identification of phosphate-solubilizing native bacteria isolated from the rhizosphere of Prosopis glandulosa in Mexicali valley. *Genetics and molecular research : GMR* **14**, 2793-2798 (2015).

3 Azziz, G. *et al.* Abundance, diversity and prospecting of culturable phosphate solubilizing bacteria on soils under crop-pasture rotations in a no-tillage regime in Uruguay. *Applied Soil Ecology* **61**, 320-326 (2012).

4 Liu, F. P. *et al.* Isolation and characterization of phosphate-solubilizing bacteria from betel nut (Areca catechu) and their effects on plant growth and phosphorus mobilization in tropical soils. *Biology And Fertility Of Soils* **50**, 927-937 (2014).

5 Anzuay, M. S. *et al.* Genetic diversity of phosphate-solubilizing peanut (Arachis hypogaea L.) associated bacteria and mechanisms involved in this ability. *Symbiosis* **60**, 143-154 (2013).

6 Singh, P., Kumar, V. & Agrawal, S. Evaluation of phytase producing bacteria for their plant growth promoting activities. *International journal of microbiology* **2014**, 426483 (2014).

7 Zhao, K. *et al.* Maize rhizosphere in Sichuan, China, hosts plant growth promoting Burkholderia cepacia with phosphate solubilizing and antifungal abilities. *Microbiol Res* **169**, 76-82 (2014).

8 Nobandegani, M. B. J., Saud, H. M. & Yun, W. M. Phylogenetic Relationship of Phosphate Solubilizing Bacteria according to 16S rRNA Genes. *Biomed Res Int* (2015).

9 Chen, Y. P. *et al.* Phosphate solubilizing bacteria from subtropical soil and their tricalcium phosphate solubilizing abilities. *Applied Soil Ecology* **34**, 33-41 (2006).

10 Ghosh, U. *et al.* Isolation and characterization of phosphate-solubilizing bacteria from seagrass rhizosphere soil. *J Ocean U China* **11**, 86-92 (2012).

11 Ndung'u-Magiroi, K. W. *et al.* Occurrence and genetic diversity of phosphate-solubilizing bacteria in soils of differing chemical characteristics in Kenya. *Ann Microbiol* **62**, 897-904 (2012).

12 Sahay, R. & Patra, D. D. Identification and performance of stress - tolerant phosphate - solubilizing bacterial isolates on Tagetes minuta grown in sodic soil. *Soil Use and Management* **29**, 494-500 (2013).

13 Acevedo, E., Galindo-Castaneda, T., Prada, F., Navia, M. & Romero, H. M. Phosphate-solubilizing microorganisms associated with the rhizosphere of oil palm (Elaeis guineensis Jacq.) in Colombia. *Applied Soil Ecology* **80**, 26-33 (2014).

14 Kwak, Y., Jung, B. K. & Shin, J. H. Complete genome sequence of Pseudomonas rhizosphaerae IH5T (=DSM 16299T), a phosphate-solubilizing rhizobacterium for bacterial biofertilizer. *Journal of biotechnology* **193**, 137-138 (2015).

15 Qin, S. W., Gu, Y. C. & Zhu, Z. L. A preliminary report on long-term stationary experiment on fertility evolution of fluvo-aquic soil and the effect of fertilization. *Acta Pedologica Sinica* **35**, 367-375 (1998).

16 Chu, H. *et al.* Soil microbial biomass, dehydrogenase activity, bacterial community structure in response to long-term fertilizer management. *Soil Biology and Biochemistry* **39**, 2971-2976 (2007).

17 Liu, G. J., Pang, H. D. & Li, Y. Y. Effects of long-term fertilization on the growth and photosynthesis of summer maize in fluvo-aquic soil. *Plant Nutrition and Fertilizer Science* **16**, 1094-1099 (2010).
